# An Inaugural Dissertation upon the Avulsion of the Arm and Scapula

**Published:** 1859-08

**Authors:** H. B. Horlbeck

**Affiliations:** Charleston, S. C.


### AN INAUGURAL DISSERTATION UPON THE AVULSION OF THE ARM AND SCAPULA.

BY H. B. HORLBECK, OF CHARLESTON, S. C.

This report includes the history of four cases of this very formidable injury, and illustrates what we should not be ready to expect, that the system submits to the rending away so important a part of the body as the arm and scapula, and the consequent laceration of muscles, nerves and vessels, with a much less sensible disturbance than we often witness after injuries that in comparison appear trivial; for in these cases there was said to be "almost entire absence of nervous shock, the harmony of the great functions of life being almost undisturbed." The explanation offered for this phenomenon is, "that all the bruised and contused tissues have been entirely removed, and only those of a sound and healthy nature left." If this is the true solution of the alleged fact, would it not argue strongly in favor of immediate amputation after severe injuries? thus including the operation and injury in a single shock, and freeing the system at once from all "contused tissue." Of these four cases, all recovered, two with, and two without ligature of the wounded artery; but, in reading an account of them, we are led to question the necessity of the use of the ligature in either case, and to think that the danger to the patient was augmented by the disturbance of the wound, which was necessary in order to secure the vessel. The explanation given of the well known fact, that a lacerated artery does not emit much blood, is clear and undoubtedly correct, and is to be sought in the anatomical formation of those vessels. When traction is put upon an artery sufficient to tear it asunder, its internal and middle coats, whose powers of assistance are slight, first give way and retract; the external coat, composed of condensed cellular issue, is elongated, and, finally parting, closes over the divided end of the middle and interior coats, and thus retains a coagulation of blood up to the point where the first vessel is given off; in this manner fulfilling in every particular the purposes of a ligature. These cases are interesting, as demonstrating the ability of the system to surmount the effect of most extensive mutilation, and will serve to put us on our guard against unnecessary interference for the purpose of ligating vessels, though they be of considerable magnitude, if they are torn across and do not bleed.—*Charleston Medical Journal.*

